# 

**Published:** 1856-07

**Authors:** 


					﻿No. II.]
JULY.
[Vol. XII,
BUFFALO
MEDICAL JOURNAL
AND
Jltotljb) tkmew
MEDICAL AND SURGICAL SCIENCE.
SANFORD B. HUNT, NI. D.,
HDITOK.
BUFFALO, N. Y.:
THOMAS <fc LATHROPS’ STEAM PRESS
Commercial Advertiser Buildings.
Price, $2.00 i» advance, or $2.50 at the end of three month*
				

